# 308-nm Excimer Lamp vs. Combination of 308-nm Excimer Lamp and 10% Liquor Carbonis Detergens in Patients With Scalp Psoriasis: A Randomized, Single-Blinded, Controlled Trial

**DOI:** 10.3389/fmed.2021.677948

**Published:** 2021-06-15

**Authors:** Ploysyne Rattanakaemakorn, Korn Triyangkulsri, Wimolsiri Iamsumang, Poonkiat Suchonwanit

**Affiliations:** Division of Dermatology, Faculty of Medicine, Ramathibodi Hospital, Mahidol University, Bangkok, Thailand

**Keywords:** excimer lamp, phototherapy, coal tar, targeted phototherapy, UVB

## Abstract

**Background:** Scalp psoriasis is usually refractory to treatment. Excimer devices have been proved to be a promising therapeutic option in psoriasis. Greater efficacy of phototherapy can be achieved by concurrent use of coal tar derivatives.

**Objective:** We aimed to compare efficacy and safety between 308-nm excimer lamp monotherapy and a combination of 308-nm excimer lamp and 10% liquor carbonis detergens in the treatment of scalp psoriasis.

**Methods:** In this randomized, evaluator-blinded, prospective, comparative study, 30 patients with scalp psoriasis received either 308-nm excimer lamp monotherapy or a combination of 308-nm excimer lamp and 10% liquor carbonis detergens twice per week until complete remission of the scalp or for a total of 30 sessions. Efficacy was evaluated by the improvement of Psoriasis Scalp Severity Index (PSSI) score, itch score, and Scalpdex score.

**Results:** Both treatments induced significant improvement in PSSI score with greater reduction observed in the combination group. At 30th visit, a 75% reduction in PSSI (PSSI75) was attained by 4 (28.6%) and 9 (69.2%) patients treated with monotherapy and combination therapy, respectively (*P* < 0.05).

**Conclusions:** Excimer lamp is well-tolerated in patients with scalp psoriasis and liquor carbonis detergens can be used as a combination therapy to improve the efficacy of excimer lamp.

## Introduction

Psoriasis is a common dermatologic disease with a prevalence of ~0.5–11% worldwide (1). It has several clinical presentations which eventually develop into chronic plaque psoriasis. The scalp is commonly affected and the frequency tends to increase with the disease duration (2). Compare to other areas of the body, the scalp is relatively refractory to many of the treatment modalities (3).

Ultraviolet (UV) radiation, both A and B, is known to be an effective treatment of psoriasis. Excimer laser and non-laser devices offer a narrow spectrum of UV light and greater localization of irradiation allowing a lower number of treatments and cumulative dose as well as sparing of uninvolved skin to produce higher efficacy (4). Earlier studies found that 308-nm excimer laser was able to achieve exceptional results in the previously recalcitrant area of the scalp (5–7). A previous study using a 308-nm excimer lamp, a non-laser device, also demonstrated a similar favorable result in the treatment of scalp psoriasis with minimal and transient side effects (8). Comparing to the excimer laser, the excimer lamp has the superior advantage of being able to give uniform irradiation of 50 times wider area in a single exposure at a lower cost (9).

Coal tar is one of the traditional treatments for psoriasis. Other than having anti-inflammatory, antibacterial, antipruritic, and antimitotic effects, coal tar is also a photosensitizer (10). Coal tar, when used together with UVB light, provides a synergistic effect with better treatment outcomes than either treatment alone (11). Goeckerman regimen is an example of the application of coal tar with phototherapy (12). The regimen requires the patient to apply coal tar to the lesion for 5 h and rinsed off before undergoing phototherapy. The process boasts a fast resolution of psoriasis with 100% of patients attained a 75% reduction in Psoriasis Area and Severity Index at ~12 weeks (13).

We hypothesized that with liquor carbonis detergens (LCD), a coal tar derivative, the treatment of excimer lamp could be enhanced to give a superior treatment outcome to the excimer lamp alone. This study aimed to compare the efficacy and safety of 308-nm excimer lamp monotherapy and 308-nm excimer lamp in combination with 10% LCD in the treatment of scalp psoriasis.

## Materials and Methods

### Study Design and Patients

This is a randomized, evaluator-blinded, controlled study of 308-nm excimer lamp as monotherapy and combination of 308-nm excimer lamp with 10% LCD in scalp psoriasis. This study was conducted as a pilot study. The sample size estimation was based on data from the previous 308-nm excimer lamp study in the Asian population. To achieve a power of 80% and a two-sided significance level of 5%, the minimum sample size required was 9 in each group (8). Thirty patients with clinically diagnosed plaque-type scalp psoriasis were enrolled in the study. The study was approved by the Committee of Human Rights Related to Research Involving Human Subjects, Mahidol University (ID 09-60-09, thaiclinicaltrials.org identifier: TCTR20171128003) and conducted in accordance with the Declaration of Helsinki. All patients provided written informed consent. Patients age 18 years or older who have been diagnosed with plaque-type psoriasis of the scalp involving at least 1% of total body surface area were included. The exclusion criteria were (i) pustular or erythrodermic psoriasis; (ii) presence of severe systemic disease; (iii) a history of photosensitivity or taking photosensitive medication; (iv) a history of skin cancer; (v) being pregnant or lactating; and (vi) allergy to any coal tar derivatives. Patients' current systemic treatments without recent modification (within 6 months) were maintained throughout the study period; however, topical agents for the scalp were required to be discontinued before enrolling in the study and until the last follow-up appointment.

Upon enrollment, a detailed history was obtained from each patient with special attention on the duration of the disease, area of involvement, as well as the history of previous therapies and current therapies. Each patient was randomly assigned using a random number table to receive either excimer lamp monotherapy or excimer lamp in combination with 10% LCD therapy (combination therapy).

### Treatment

The 308-nm excimer lamp (Therabeam^®^ UV308, Ushio Inc., Tokyo, Japan) was used for both groups. Treatment was performed twice per week. Each patient was treated for 30 sessions, or until complete clearing of the scalp occurred. Beginning with 500 mJ/cm^2^ for all patients, we increased the irradiation dose by 10% every treatment during the whole treatment period. The irradiation dose was fixed when clinically noticeable improvement was observed. If severe side effects including blistering, burn or severe pain occurred, the treatment was skipped until complications subsided. The treatment would then resume with the dose that did not cause any side effects on the subsequent visit. Participants who failed to attend treatment for more than 3 weeks consecutively were excluded. Patients in the combination therapy group were additionally asked to apply 10% LCD cream efficiently throughout the plaques on their scalp for at least 5 h or overnight and rinsed off before each treatment session.

### Assessment

At baseline, 20th visit, 30th visit, and 4 weeks after the last treatment, Psoriasis Scalp Severity Index (PSSI) score was assessed by a blinded dermatologist. The PSSI score is calculated by assessing erythema, scaliness, and induration with a score of 0–4 for each symptom. The extent of scalp psoriasis involvement ranging from 0 to 6 is then calculated and multiplied with the score of the symptoms resulting in a total score of 0–72 (14).

Patients were requested to rate their scalp-related itch and Scalpdex score at baseline, 20th visit, 30th visit, and after last treatment. Itch score was rated using a 0–10 scale, with higher scores indicating greater severity. Scalpdex score requires the patients to rate frequency of impact for 23 scalp-related items using a 0–100 scale with 0 = never, 25 = rarely, 50 = sometimes, 75 = often, and 100 = all the time. The items are categorized into symptoms, functioning, and emotions. Higher scores indicate greater impairment of quality of life in each aspect (15).

### Statistical Methods

Data were analyzed using STATA/SE version 14.2 (STATA Corp., College Station, TX). Categorical variables were expressed as percentages and were analyzed using either the chi-squared test or Fisher's exact-test. Continuous variables were expressed in terms of either mean (standard deviation) for normally distributed variables or median (range) for non-normal distributed variables and were evaluated using a mixed model. A *P*-value of <0.05 was considered statistically significant.

## Results

### Patient Characteristics

Thirty patients (13 males, 17 females; age 21–72, mean age 41 years) were enrolled in this study and were randomly divided into 2 groups. There was a significant difference in mean age between the two groups. Other baseline demographics were similar between treatment groups (Table 1). Twenty-seven patients completed the study while 3 were excluded because of their inability to adhere to treatment frequency due to personal or unforeseen circumstances. Baseline disease characteristics after excluding 3 patients displayed some but not statistically significant difference in median baseline PSSI. There was also a significant difference in median baseline itch score after excluding 3 patients. Thus, these 2 variables (age and itch score) were adjusted in the statistical analysis. Baseline Scalpdex scores were similar between treatment groups (Table 2).

**Table 1:** Demographics and characteristics of the patients at baseline.

**Characteristics**	**Monotherapy**	**Combination**	***P-*value**
	**(*N* = 15)**	**therapy (*N* = 15)**	
**Sex**; *N* (%)			0.713
Male	6 (40.0%)	7 (46.7%)	
Female	9 (60.0%)	8 (53.3%)	
**Age** in year; mean (SD)	47 (15.0)	35.53 (12.7)	0.032^*^
**BMI** in kg/m^2^; mean (SD)	29.10 (6.5)	27.15 (5.5)	0.386
**Fitzpatrick skin type**; *N* (%)			0.705
III	9 (60.0%)	10 (66.7%)	
IV	6 (40.0%)	5 (33.3%)	
**Onset** in years; mean (SD)	33.92 (13.9)	27.63 (11.1)	0.182
**Duration** in years; median (range)	9 (0.25–40)	5 (0.5–29)	0.228
**Family history**; *N* (%)			0.682
Yes	3 (20.0%)	5 (33.3%)	
No	12 (80.0%)	10 (66.7%)	
**Psoriatic arthritis**; *N* (%)			0.682
Yes	5 (33.3%)	3 (20.0%)	
No	10 (66.7%)	12 (80.0%)	
**Current systemic treatment**; *N* (%)			0.700
Yes	6 (40.0%)	4 (26.7%)	
No	9 (60.0%)	11 (73.3%)	
**Effective dose** in mJ/cm^2^; mean (SD)	1,364.3 (315.9)	1,165.4 (315.9)	0.134

*BMI, Body mass index*.

* *Statistically significant*.

**Table 2:** Treatment results on patients throughout the study duration.

**Data**	**Monotherapy**	**Combination**	***P-*value**
	**(*N* = 14)**	**therapy (*N* = 13)**	
**PSSI; median (range)**			
Baseline	18 (8–30)	12 (3–30)	0.149
20th visit	12.5 (2–25)	3 (0–6)	0.021^*^
30th visit	12 (0–25)	3 (0–4)	0.016^*^
4 weeks after last treatment	8 (0–20)	3 (0–4)	0.022^*^
**Itch score; median (range)**			
Baseline	7 (2–8)	4.5 (0–9)	0.050^*^
20th visit	4 (0–8)	2 (0–5)	0.316
30th visit	2 (0–7)	2 (0–7)	0.515
4 weeks after last treatment	2 (1–7)	3 (0–7)	0.175
**Scalpdex total score; median (range)**			
Baseline	39.7 (6.5–80.4)	42.4 (30.4–73.9)	0.961
20th visit	17.9 (4.3–88.0)	14.1 (3.3–45.7)	0.213
30th visit	17.4 (0.0–82.6)	16.3 (1.1–44.6)	0.256
4 weeks after last treatment	15.2 (2.2–90.2)	9.2 (0.0–64.1)	0.403

*PSSI, Psoriasis Scalp Severity Index*.

* *Statistically significant*.

### Efficacy

**Figure 1:**
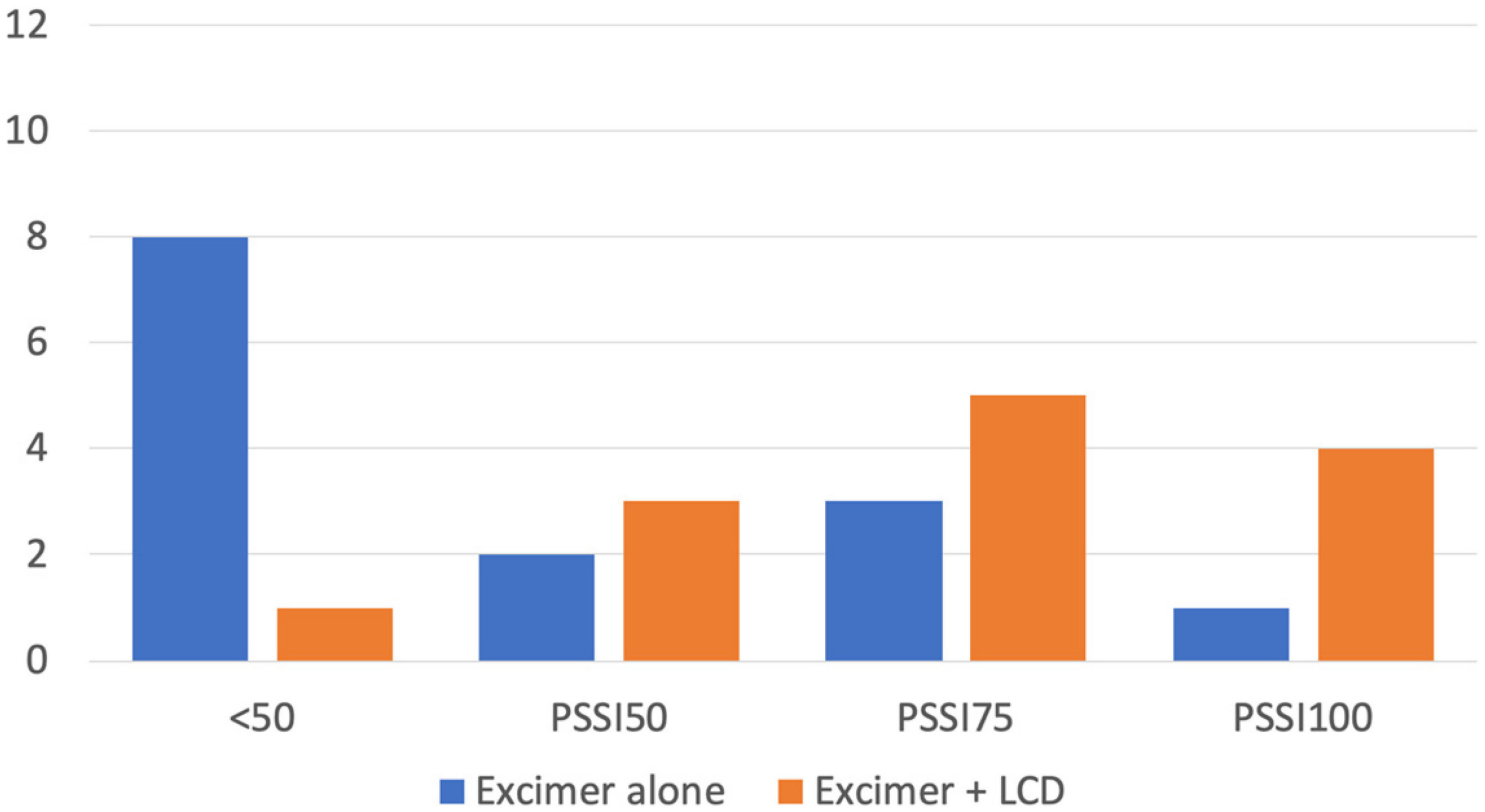
Number of patients achieving clearance of various percentages at 30th visit. Patients achieving <50% clearance (<PSSI50), 50% clearance (PSSI50), 75% clearance PSSI75, and 100% clearance (PSSI100). *PSSI*, Psoriasis Scalp Severity Index; *LCD*, Liquor carbonis detergens.

**Figure 2:**
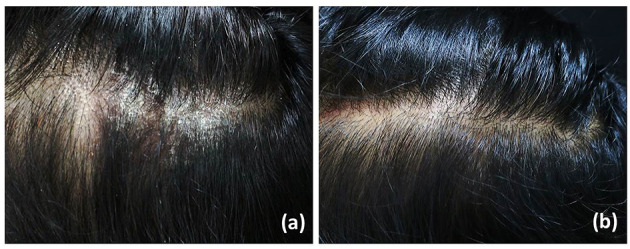
Psoriatic plaque, before **(a)** and after **(b)** treatment with excimer lamp and 10% LCD cream at 30th visit.

Both treatment groups achieved a significant reduction in PSSI score. In the monotherapy group, the median PSSI score was reduced from 18 (8–30) to 12.5 (2–25) at 20th visit, 12 (0–25) at 30th visit, and 8 (0–20) at 4 weeks after the last treatment (*P* < 0.001). Similarly, the combination therapy group's median PSSI score was reduced from 10.5 (3–24) to 3 (0–6) at 20th visit, 3 (0–4) at 30th visit, and 3 (0–4) at 4 weeks after the last treatment (*P* < 0.001). The combination therapy group was able to achieve a significantly greater reduction in PSSI score than the monotherapy group at every assessment time (*P* = 0.001; Table 2). At 30th visit, the combination therapy group had a larger percentage of patients reaching PSSI50, PSSI75, or PSSI100 than the monotherapy group (*P* < 0.05) (Figures 1, 2). However, by 4 weeks after treatment cessation, monotherapy group had more patients who continued to improve (PSSI50 = 46.1%, PSSI75 = 23.1%, PSSI100 = 7.7%) resulting in a more similar achievement to combination therapy group (PSSI50 = 25.0%, PSSI75 = 41.7%, PSSI100 = 25.0%) (*P* = 0.362). In terms of itch score, both treatment groups were found to have a significant reduction (*P* < 0.05) but there was no significant difference between the two (*P* = 0.597). Overall Scalpdex score displayed significant reduction in both groups (*P* < 0.001) without significant difference between groups (*P* = 0.366; Table 2).

The monotherapy group seemed to require a slightly higher irradiation dose than the combination therapy group at a mean effective dose of 1364.3 (±315.9) mJ/cm^2^ and 1165.4 (±315.9) mJ/cm^2^, respectively (*P* = 0.134). Mean cumulative dose at 30th visit demonstrated a similar pattern as the monotherapy group having 32702.9 (±3997.3) mJ/cm^2^ while the combination therapy group having 27779.2 (±8860.9) mJ/cm^2^ (*P* = 0.101).

### Safety

Common adverse events that occurred in both groups were itch and pain after the treatment, which resolve spontaneously without any treatment within 1–2 days. No patient experienced any pain or discomfort during the treatment. Five patients (35.7%) from the monotherapy group developed blisters compared to 1 patient (8.3%) from the combination therapy group (*P* = 0.170). The first-degree burn was observed in 1 patient from each group. The combination therapy group had severe adverse events observed at a lower mean dose of 680 (±28.3) mJ/cm^2^ when compared to the monotherapy group, 1,180 (±345.7) mJ/cm^2^ (*P* = 0.111). No patient dropped out due to adverse events.

## Discussion

Immunomodulation is the key therapeutic mechanism of phototherapy in psoriasis. Phototherapy interfered with antigen presentation of Langerhans cells to the T cell which in turn affects cytokines and adhesion molecules that are overexpressed in psoriatic plaques (16, 17). It also downregulates Th17 expression, cytokine expression, and causes a shift in cytokine profiles from a Th1 to a Th2 response (18, 19). By interposing with the synthesis of proteins and nucleic acids, UV radiation also inhibits epidermal hyperproliferation and angiogenesis (20–22). Various UVB sources with wavelength ranging from 290 to 320 nm are commonly used in the treatment of psoriasis. Among these, excimer devices that are able to produce a spectrum of 308 nm radiation have been shown to be efficacious in treating psoriatic plaques. A study demonstrated the efficacy of a single high dose 308-nm excimer laser treatment, clearing psoriasis plaque (23). An immunohistochemical study found that psoriatic skin after excimer light therapy showed significant T-cell depletion and alterations of apoptosis-related molecules associated with a decreased proliferation index and clinical remission (24). The excimer lamp irradiation also shows an antipruritic effect *via* induction of epidermal nerve degeneration (25).

In this study, the excimer lamp alone is efficacious and well-tolerated for scalp psoriasis whereas LCD cream was shown to enhance its efficacy without a significant increase in adverse events. Given many of the patients in our study were considered refractory to the ongoing treatment, they reportedly achieved improvement after excimer lamp treatment with or without LCD cream. Additionally, the effects of both treatment regimens were maintained up to 4 weeks after the last treatment. Furthermore, the monotherapy group showed a higher number of patients with ongoing improvement. We hypothesize that UVB phototherapy could induce a long remission period by promoting apoptosis of pathologically relevant T cells, especially tissue-resident memory T cells (26, 27). A higher cumulative irradiation dose used in the monotherapy group may result in more patients with continuous improvement. However, concurrent application of 10% LCD cream also resulted in less irradiation effective dose, therefore hastens reduction rate of PSSI score. This can result in less long-term cumulative UV exposure. The author would also like to point out that in this study, 10% LCD cream was only used on the night before excimer lamp treatment for its photodynamic property and thus effects of the treatment may be enhanced further if 10% LCD cream was applied regularly or more frequently.

The main setbacks of 10% LCD cream are its unfavorable smell, its ability to readily stain onto fabric material, and possible contact dermatitis. Lastly, usage of LCD cream or other coal tar derivatives can interfere with UV transmission and should be removed thoroughly before exposure to phototherapy (28–32). Although the detail of photodynamic activity of coal tar was still unclear and only proven with an action spectrum in UVA and visible light (33, 34), several studies had demonstrated the effectiveness of coal tar in enhancing the therapeutic outcome of UVB spectrum treatment similar to our study suggesting rooms for further research in elucidating the actual mechanism and possible light spectrum range for UVB of coal tar photodynamic activity (13, 35–37).

A previous study of excimer lamp showed that 6 out of 28 patients (30%) were able to achieve PSSI75 after only 10 sessions and 5 patients (25%) achieved PSSI50 (8). These numbers showed a favorable result of excimer lamp similar to this study. However, the treatment sessions required were much shorter than our study which we suspect to be due to the patient's concurrent treatment of topical medication. A previous study evaluating excimer laser found that the majority of patients (56.52%) achieved PSSI75 while 34.78% of patients were able to achieve PSSI50 at 24th visit (7). These results triumph over our monotherapy group. However, our combination therapy group attained comparable improvement at 30th visit (15 weeks), accounting for 69.2 and 23.1% for PSSI75 and PSSI50, respectively. Furthermore, it is important to note that among 69.2% with PSSI75, 4 patients (30.7%) achieved PSSI100.

As for safety issues, the monotherapy group showed a higher incidence of adverse events due to the higher irradiation dose used. Nevertheless, dose adjustment was able to prevent the reoccurrence of the adverse events. Blistering was seen mainly when the dose was higher than 1,100 mJ/cm^2^ and readily resolve spontaneously or with a short course of moderate potency topical corticosteroid within 7–10 days. Similar case series documenting cases with blistering after narrowband UVB therapy were able to continue and complete the treatment course with lowered irradiation dose. These cases too were able to complete their phototherapy and the occurrence of blisters subsided after topical corticosteroid treatment and dose adjustment as well (38). Few studies using excimer devices both light and laser had reported some patients with blistering (6, 7, 39). This suggests that blistering might just be due to too high irradiation dose. However, this also proved that patients can tolerate excimer lamp at a much lower dose than narrowband UVB in general. Therefore, attention must be paid to dose adjustment and increment while using excimer devices. Safety of having concurrent vitamin A derivatives intake was not addressed in our study as they were in the exclusion criteria.

The limitations of this study include a limited number of patients and a relatively short follow-up period after treatment cessation. Although the assignments were randomized, there was a significant difference in baseline severity of scalp psoriasis between the two groups. The monotherapy group had more severe baseline disease, which might contribute to their lower response rate. Future studies involving larger populations and longer study duration are warranted in elucidating long-term safety and remission time.

## Conclusion

Combination therapy of excimer lamp and 10% LCD showed promising results with 92.3% of patients achieving PSSI50 and above with minimal and reversible adverse events. Concerning scalp psoriasis, the combination of excimer lamp therapy and 10% LCD is highly efficacious and well-tolerated.

## Data Availability Statement

The raw data supporting the conclusions of this article will be made available by the authors, without undue reservation.

## Ethics Statement

The studies involving human participants were reviewed and approved by the Committee of Human Rights Related to Research Involving Human Subjects, Mahidol University. The patients/participants provided their written informed consent to participate in this study.

## Author Contributions

PS: conceptualization. PS and PR: methodology, validation, and writing–review and editing. KT and WI: formal analysis and data curation. PS, KT, and WI: investigation. KT and PR: writing–original draft preparation. All authors have read and agreed to the published version of the manuscript.

## Conflict of Interest

The authors declare that the research was conducted in the absence of any commercial or financial relationships that could be construed as a potential conflict of interest.
